# Comprehensive traditional East Asian medicine treatment strategy for obesity considering the therapeutic effects and adverse events: A protocol for systematic review and network meta-analysis

**DOI:** 10.1097/MD.0000000000028673

**Published:** 2022-02-11

**Authors:** Hongmin Chu, Byungsoo Kang, Bo-Young Youn, Kwan-il Kim, Jinbong Park, Jungtae Leem

**Affiliations:** a Daecheong Public Health Subcenter, 3, Daecheong-ro, Daecheong-myeon, Ongjin-gun, Incheon, Republic of Korea; b College of Korean Medicine, Dongshin University, 67, Dongsindae-gil, Naju-si, Jeollanamdo, Republic of Korea; c Department of Preventive Medicine, College of Korean Medicine, Kyung Hee University, 26, Kyungheedae-ro, Seoul, Republic of Korea; d Division of Allergy, Immune Division of Allergy, Immune and Respiratory System, Department of Internal Medicine, College of Korean Medicine, Kyung Hee University, 23 Kyungheedae-ro, Dongdaemun-gu, Seoul, Republic of Korea; e Department of Pharmacology, College of Korean Medicine, Kyung Hee University, 26 Kyungheedae-ro, Dongdaemun-gu, Seoul, Republic of Korea; f Research Center of Traditional Korean Medicine, Wonkwang University, 460, Iksan-daero, Sin-dong, Iksan, Jeollabuk-do, Republic of Korea.

**Keywords:** acupuncture, herbal medicine, network meta-analysis, obesity, systematic review, traditional East Asian medicine

## Abstract

**Background:**

Obesity has become a serious global health problem due to its increasing prevalence. Because of several limitations or adverse events associated with conventional western medicine therapies, there has been an increase in demand for alternative therapies such as traditional East Asian medicine (TEAM). This study aims to provide comprehensive evidence-based information assessing the clinical efficacy and safety of TEAM treatment for obesity as the basis for reliable clinical strategies for patients with obesity.

**Methods:**

Electronic searches of the PubMed, Cochrane Library, EMBASE, China National Knowledge Infrastructure, OASIS, and Korea Citation Index will be performed. Methodological quality will be assessed using the “risk of bias” tool. The primary outcome for efficacy will be weight loss. The secondary outcomes will be response rate, body mass index, waist circumference, and blood pressure. We will also evaluate the rates of adverse events and mortality for safety assessment. First, we will conduct a conventional pairwise meta-analysis. Next, we will conduct network meta-analysis using the frequentist approach. We shall verify the assumption of network meta-analysis and provide network geometry, P-score, net league table, and intervention-based forest plot. A subgroup analysis will be conducted to ascertain the factors that affect treatment, such as dosage, treatment duration, and severity of obesity.

**Results:**

The results of this study will provide high-quality systematic reviews that can assist decision making in obesity management. Our network meta-analysis results can provide direct and indirect comparison evidence on comparative efficacy and safety.

**Conclusion:**

This study will provide fundamental data for prospective research on the application of TEAM in patients with obesity.

**Protocol registry number of online registry:**

This study protocol was registered in open Science framework (OSF) (Registration DOI: 10.17605/OSF.IO/ETWDS)

**URL of the online registry:**

https://osf.io/etwds.

## 1 Introduction

Obesity is a disease in which an abnormally excessive amount of fat is accumulated in the body.^[1]^ It can be caused by lifestyle problems such as excessive calorie intake or a lack of exercise.^[2]^ According to the World Health Organization, it is estimated that 39% of the worldwide adult population will become obese by 2035.^[3]^ Obesity is especially prevalent in East Asia, where the diet has become westernized due to rapid economic growth and lifestyle changes.^[3]^ Overweight and obesity rates have risen dramatically in most Asian countries over the last few decades, with varying degrees of severity.^[4–6]^ Obesity not only threatens physical health but also psychological health, reducing the quality of life of individuals. Therefore, various pharmacological and non-pharmacological treatments for the management of obesity are being tested.^[7]^ Obesity is also a risk factor for various metabolic, cardiovascular, and cerebrovascular diseases.^[2]^ Thus, as the obese population increases, the overall health status of the society worsens and its socioeconomic burden increases.^[4]^ In this respect, along with the rise in obese population, the obesity treatment market has been steadily increasing. By 2021, the global cost of obesity treatment will reach 2 trillion dollars.^[8]^

There are various methods for obesity management. Pharmacological therapies with conventional drugs such as phentermine and orlistat, as well as surgical treatments, are widely used.^[9]^ In addition, there have recently been other treatments such as behavioral correction using digital therapeutics.^[10]^ However, there are some problems associated with existing obesity treatments. Liposuction may cause gastrointestinal hemorrhage, skin necrosis, or a severe bruise after surgery.^[11,12]^ Phentermine may lead to cardiovascular diseases such as pulmonary arterial hypertension, while orlistat may cause gastrointestinal side effects such as steatorrhea and diarrhea.^[7]^ Some drugs that were used to treat obesity have been withdrawn due to the risk of severe adverse events. Regarding sibutramine, a rebound in body weight occurs after stopping the medication, and headache, dry mouth, insomnia, and constipation are its common side effects.^[13,14]^ Moreover, cognitive behavioral therapies for obesity have limited effectiveness in promoting long-term weight loss.^[15]^ In surgical treatment, iron deficiency anemia and reoperations were reported as the most common adverse events.^[16]^

Therefore, there is an increasing demand for an alternative therapeutic strategy for obesity treatment. In East Asia, various traditional medicine interventions are used for obesity treatment.^[17–19]^ Traditional East Asian medicine (TEAM) treatment for obesity management not only includes pharmacological (traditional herbal medicine) therapy, but also acupuncture, electro-acupuncture, pharmacopuncture, moxibustion, Qi-gong, cupping therapy, and other methods.^[9,20–24]^ Herbal medicine using Ephedra sinica is a pivotal intervention that affects obesity-related clinical indicators such as weight, body fat mass, and abdominal circumference.^[25–27]^

However, it is unknown which of the TEAM treatments is effective for weight loss while having the least adverse events. Recently, a research methodology called network meta-analysis (NMA), which explores the effects of various interventions using network analysis techniques, has been used for a head-to-head comparison of various therapies.^[28]^ This study aims to provide comprehensive evidence-based information assessing the clinical efficacy and safety of TEAM treatment for obesity as the basis for reliable clinical strategies for patients with obesity. In obesity management, controlling adverse events is an important clinical issue for consistent treatment. Therefore, we will also assess safety based on adherence (drop-out rate) and adverse events.

## 2 Materials and methods

We followed the Preferred Reporting Items for Systematic reviews and Meta-Analyses for NMA (PRISMA-NMA, Supplemental Digital Content) checklist.^[29]^

### 2.1 Protocol registration and ethics

This systematic review protocol was registered in open Science framework (OSF) (Registration DOI: 10.17605/OSF.IO/ETWDS; URL: https://osf.io/etwds) and followed the guidelines mentioned in the PRISMA statement.^[29]^ Since this study will quantitatively synthesize data from previously published papers, institutional review board approval and consent of the subject are not required.

### 2.2 Data sources and search methods

The following six databases were searched from their inception to July 2021: Medline (PubMed, https://pubmed.ncbi.nlm.nih.gov/), Cochrane Library (CENTRAL, https://www.cochranelibrary.com/), EMBASE (https://www.embase.com), OASIS (https://oasis.kiom.re.kr/), Korea Citation Index (https://www.kci.go.kr), and China National Knowledge Infrastructure (https://oversea.cnki.net/index/). The search strategies for Medline (via PubMed) database are shown in Table 1.

**Table 1:** Search strategy for medline (via PubMed).

#1	Search obesity[Title/Abstract]
#2	Search Weight loss [Title/Abstract]
#3	Search Over weight[Title/Abstract]
#3	Search obes^∗^ or “body mass ind^∗^” or adipos^∗^ or overweight or “over weight” or “overload syndrom^∗^” or overeat^∗^ or “over eat^∗^” or overfeed^∗^ or “over feed^∗^” or overfed or “over fed” or "weight cycling” [Title/Abstract]
#4	Search antiobesity or “anti- obesity” or obesitas or bodyweight or “body weight” [Title/Abstract]
#5	Search #1 OR #2 OR #3 OR #4
#6	Search traditional Chinese medicine[MeSH Terms]
#7	Search Drugs, Chinese Herbal[MeSH Terms]
#8	Search Korean Medicine
#9	Search traditional Chinese herbal medicine[Title/Abstract]
#10	Search Kampo^∗^[Title/Abstract]
#11	Search Chinese herb^∗^[Title/Abstract]
#12	Search #7 OR #8 OR #9 OR #10 OR #11
#12	Search #5 AND #12

### 2.3 Inclusion criteria

#### 2.3.1 Participants

We will include studies for the general adult human population that have evaluated overweight or obese individuals (body mass index [BMI] ≥ 25).^[30]^ Studies on secondary obesity caused by specific diseases or conditions will be excluded.

#### 2.3.2 Types of studies

This systematic review will include randomized controlled trials (RCTs) and the first phase of cross-over studies. Observational studies, case series, case reports, and ecological studies will be excluded. Furthermore, there will be no language restrictions. Articles which do not provide full text will also be excluded.

#### 2.3.3 Interventions and comparators

In the primary phase, we will review the titles and abstracts and select suitable studies. Next, the full texts of the selected studies will be reviewed, and a final decision will be made on whether or not to include the article. RCTs on obesity treated with TEAM interventions such as herbal medicine, acupuncture, pharmacopuncture, electro-acupuncture, moxibustion, cupping therapy, manual therapy, and qi-gong will be included. RCTs on other complementary and alternative medicine (CAM) interventions (excluding TEAM interventions) such as Yoga or neurofeedback therapy will be excluded. The types of TEAM interventions were selected by referring to previous literature.^[31,32]^ General rehabilitation or exercise therapy are not of interest in our study.

#### 2.3.4 Types of outcomes

1. Primary outcome for efficacy: weight loss (body weight)^[33]^
2. Primary outcome for safety: rate of adverse events
3. Secondary outcomes for efficacy: The percentage of participants who lose more than 5% of their body weight, BMI, waist circumference, blood pressure, blood glucose, blood lipids, health-related quality of life, and psychological evaluation.^[34,35]^
4. Secondary outcomes for safety: Drop-out ratio and all-cause mortality.

#### 2.3.5 Study design

1. Studies involving combination therapy of TEAM will not be included in the systematic review. For example, comparative trials of the acupuncture and herbal medicine combination treatment group and the herbal medicine single treatment group will not be included in this research.
2. In the control group, only conventional western medicine therapy groups or placebo controls will be included. However, placebo controls will be divided into pharmacological placebo (placebo herbal medicine) control and physiological placebo (placebo acupuncture and sham acupuncture) control groups.
3. Add-on study designs will be excluded, and only studies with a head-to-head comparison design will be included. The reason for a head-to-head comparison is that in the real world, in the clinical field of obesity treatment, due to side effects, a single treatment is mainly performed rather than a combination of several treatments.

### 2.4 Data extraction and assessment using the “risk of bias” tool

#### 2.4.1 Data extraction and assessment

The PICOS (participant, intervention, comparison, and study design) principle was used in the study design. Two authors will independently conduct and cross-check the study selection and data extraction processes (CHM and LJT). EndNote X9 (EndNote version X9, Thomson Reuters, CA) will be used to import and handle all of the retrieved literature citations. The first author, country, year of publication, number of centers, and participants, study design, number of groups, allocation ratio, interventions, comparisons, results (primary and secondary outcomes), and conclusions will all be extracted using a standard pre-designed form. A detailed treatment regimen, such as dosage, frequency, treatment duration, and individualized modification, will be also extracted. Disagreements between the 2 reviewers will be resolved through discussion or by bringing in a third reviewer (KBS).

#### 2.4.2 Risk of bias

The “risk of bias” evaluation tool, which focuses on 6 domains such as sequence generation, allocation concealment, blinding, inadequate data, selective reporting, and other bias, will be used.^[36]^ Random sequence generation, allocation concealment, blinding of participants and personnel, blinding of outcome assessment, insufficient outcome data (incomplete outcome data), and selective reporting items will be evaluated as low risk, high risk, and unclear risk.

Randomization will be evaluated as “low” when an appropriate randomization method is used, “high” when a randomization method is not used or an inappropriate method is used, and “uncertain” when the applied randomization method is unknown. Allocation concealment will be rated as “low” if the assignment order is sufficiently concealed and the researcher is unaware of the assignment, “high” if the assignment order is not sufficiently concealed or the method is inappropriate, and “uncertain” if the assignment order is unclear or the method is not specifically described. Blinding for outcome evaluation will be evaluated as “low” when blinding is performed well, “high” when blinding is not implemented or inadequate, and “uncertain” when blinding is uncertain or unknown. In case of inadequate data, if there are no missing values or if the missing values have no effect on the results, it will be rated as “low.” If there are more than 10% missing values or if the missing values affect the results, it will be rated as “high.” Insufficient reporting of missing values will be evaluated as “uncertain.” Selective reporting will be assessed as “low” when a study protocol is provided or all of the pre-planned results are reported in the paper. If the protocol is not provided or no outcomes are reported according to the determined method, it will be evaluated as “high.”

### 2.5 Data synthesis

#### 2.5.1 Geometry of the network

The R program's (R version 4.0.3, Foundation for Statistical Computing, Vienna, Austria) “forest.netmeta” function will be used to build network plots to describe and display the geometry of various treatment interventions. The nodes and edges will be utilized to show head-to-head comparisons between interventions.

#### 2.5.2 Pairwise meta-analysis

Excel 2010 will be used to summarize and present the extracted data from all of the included research. The Review Manager (RevMan) program (version 5.4, The Cochrane Collaboration, 2020) will be used to perform pairwise meta-analysis. The Higgins *I*^2^ statistic will be used to measure the statistical heterogeneity among the included studies (large if *I*^2^ > 50%; medium if 25% < *I*^2^ ≥ 50%; and small if 0 ≤ *I*^2^ ≥ 25%). If there is no evidence of heterogeneity, fixed-effect model analysis will be performed; otherwise, random-effects model analysis will be performed after the causes of heterogeneity have been eliminated.^[37]^

#### 2.5.3 NMA

An NMA based on the frequentist approach will be performed using the R software's “netmeta” function to synthesize direct and indirect comparison results for assessing the therapeutic effectiveness and safety of different therapies for obesity. If a loop connecting more than three arms exists, the node splitting method will be used to analyze inconsistency between direct and indirect comparisons using global and local approaches. P-scores, which are based on the point estimates and standard errors (SEs) of network assessment, will be used to rank the efficacy of treatment interventions for obesity patients.^[38]^

##### 2.5.3.1 Main assumptions of the NMA

NMA is based on four main assumptions: connectivity, homogeneity, transitivity, and consistency. A network plot will be used for visual inspection of connectivity of the included studies in the network. The Cochrane Q statistic or the *I*^2^ score will be used to determine homogeneity. We will also assess homogeneity qualitatively by describing the characteristics of the included studies. Because of the variability of interventions, we will use a random-effects model in our research. It is vital to investigate the distribution of effect modifiers and their impacts on the effect size while analyzing transitivity.^[39,40]^ For transitivity assessment, we will qualitatively evaluate the sample size, age, sex, disease duration, severity, treatment dosage, and treatment period. Consistency is a quantitative statistical evaluation method of transitivity, and the net splitting method will be used to assess it.

##### 2.5.3.2 Statistical assessment

We will examine both global (network level) and local approaches (particular contrast of intervention level) for consistency assumption. For the global approach, the “decomp.design” function of R software will be used to test consistency under the premise of a comprehensive design-by-treatment interaction random-effects model, and Q statistics will be used to assess inconsistency. If the *P* value for the Q statistics is less than .05, we will presume that the global network has significant inconsistency (disagreement). For the local approach, using the Facenetsplit function of R software, we will use the net splitting method to split the network estimation of effect size on each intervention into direct and indirect evidence. It will determine the difference between direct and indirect evidence and evaluate whether the difference is statistically significant. For visual inspection of inconsistencies between direct and indirect comparisons, net split plots will be provided, and if the *P* value for the net split analysis is less than .05, we will presume that a specific local loop has significant inconsistency (disagreement), indicating a significant discrepancy between direct and indirect impact size estimation. When there are significant disagreements based on specific studies, we will conduct a sensitivity analysis by sequentially excluding studies one by one. If we can identify which studies were inconsistent, we will exclude them from the NMA. In addition, a net league table will be provided. The effect size calculated by a simple direct comparison, which is similar to the pairwise comparison, will be shown in the upper right triangle. There will be multiple blanks in the upper triangle because direct comparison is not possible in all treatment comparisons. The lower left triangle will be a pooled estimate of direct and indirect effect comparisons.

### 2.6 Sensitivity and subgroup analyses

In sensitivity analysis, NMA will be performed by systematically checking each study one by one to see if any of them had a significant impact on the overall findings. The results will be visually and statistically analyzed to see whether they show consistency with the overall trend.

In another sensitivity analysis, studies with all risk of bias items checked as high risk will be excluded from the analysis. This will determine whether the quality of the research methodology affects the results or whether it is robust.

For subgroup analysis, groups with BMI > 25 or 30 will be separately analyzed. In addition, if there are sufficient studies for sub-group analysis, we will investigate factors affecting treatment effect through analysis based on dosage, duration of treatments, or whether individualized treatments or symptom classifications of TEAM were used via subgroup analysis.

### 2.7 Publication bias

Egger's test will be conducted to evaluate publication bias, and the results will be visualized using a funnel plot. Egger's test evaluates the relationship between a standardized effect estimate and the SE using linear regression.^[41]^

## 3 Discussion

This study will be the first NMA research on the treatment of obesity using TEAM. We believe that the results of this study will help physicians in deciding the TEAM intervention to be used in clinical settings based on the efficacy and safety.

The NMA method can synthesize the effect sizes of several studies with multiple interventions or treatments.^[42,43]^ NMA is also known as multiple treatment meta-analysis or mixed treatment comparison. Because this method has the advantage of comparing the size of the treatment effect for various treatments, it has recently gained popularity. In addition, the application of the NMA method is expanding in various CAM researches.^[44–47]^ Hence, to our knowledge, there have been no NMA systematic reviews comparing all TEAM interventions, especially on obesity.

Rimonabant has been associated with psychiatric disorders, whereas orlistat has been associated with gastrointestinal disorders such as defecation, fecal incontinence, fatty stools, and fecal urgency.^[48,49]^ Liposuction is associated with a risk of gastrointestinal hemorrhage, skin necrosis, and severe bruise after surgery.^[11,12]^ Concerns about these side effects and the need for alternative therapies are the reasons why the demand for TEAM therapies is gradually increasing in obesity treatment. The mechanisms of action of acupuncture in obesity are known to affect the regulation of appetite from the perspective of hypothalamus or a decrease in the expression of leptin and insulin.^[50]^ In herbal medicine, the multi-target, synergistic effect of herbs affects obesity by regulating fat metabolism, hormone level, and energy metabolism through intestinal microflora.^[51]^ In the case of ephedra, ephedrine-type alkaloids such as ephedrine, pseudoephedrine, methylephedrine, methylpseudoephedrine, and norephedrine are the main components that stimulate the α sympathetic nerve for the hypothalamic ventromedial nucleus and lateral hypothalamus. Furthermore, ephedrine indirectly suppresses appetite through dopamine activity and promotes energy consumption by increasing thermogenesis in brown adipocytes through β-sympathetic nerve stimulation. In addition, it has inhibitory effects on lipid accumulation and adipogenesis in white adipocytes. This is why ephedra sinica is used as a primary component in anti-obesity herbal medicine.^[52,53]^ In addition, since medicinal herbs such as Phellodendri cortex, Poncirus trifoliata, and fruits of Gardenia jasminoides also have inhibitory effects on lipid accumulation and adipogenesis in white adipocytes, they are being used as natural products with anti-obesity effects.^[54]^

With an increase in the number of papers on TEAM therapies for obese patients in recent years, several systematic reviews have been conducted; however, these were conducted on a single intervention or treatment, making it impossible to compare the effects of therapies.^[18,20,21,23,26]^ In particular, it is important to consider safety, tolerance, and side effects when selecting obesity therapies. High-quality systematic reviews can help provide the best evidence in clinical practice, and an NMA can provide a ranking based on comparative efficacy and safety.

The expected limitation of this study is that it is not possible to analyze the effects of complex TEAM treatments in this study. We will compensate for this limitation through follow-up studies in the future. Additional studies applying a research method such as meta-regression will be needed to identify other factors that can influence obesity management, such as treatment time, age, obesity severity, treatment duration, and gender. We devised and designed this research protocol to focus on NMA of the difference in the therapeutic effect of a single TEAM intervention. We also expect that the findings of this study will provide a new insight into obesity treatment in the CAM field, which may impact stakeholders such as clinical physicians, patients, and policy-makers in making better decisions. Furthermore, it may provide fundamental data for prospective research on the use of TEAM in patients with obesity.

## Author contributions

**Conceptualization:** Hongmin Chu, Jungtae Leem.

**Methodology:** Kwan-il Kim, Jinbong Park, Jungtae Leem.

**Supervision:** Byungsoo Kang, Bo-Young Youn.

**Writing – original draft:** Hongmin Chu.

**Writing – review & editing:** Jungtae Leem.

## Supplementary Material

Supplemental Digital Content
